# Neuropsychiatric profiles and conversion to dementia in mild cognitive impairment, a latent class analysis

**DOI:** 10.1038/s41598-021-83126-y

**Published:** 2021-03-19

**Authors:** Natalia Roberto, Maria J. Portella, Marta Marquié, Montserrat Alegret, Isabel Hernández, Ana Mauleón, Maitee Rosende-Roca, Carla Abdelnour, Ester Esteban de Antonio, Silvia Gil, Juan P. Tartari, Liliana Vargas, Ana Espinosa, Gemma Ortega, Alba Pérez-Cordón, Ángela Sanabria, Adelina Orellana, Itziar de Rojas, Sonia Moreno-Grau, Laura Montrreal, Emilio Alarcón-Martín, Agustín Ruíz, Lluís Tárraga, Mercè Boada, Sergi Valero

**Affiliations:** 1 Research Center and Memory Clinic, Fundació ACE, Barcelona Alzheimer Treatment and Research Centre, Institut Català de Neurociències Aplicades, Universitat Internacional de Catalunya (UIC) - Barcelona, Gran Vía Carles III, 85 bis, bajos, 08028 Barcelona, Spain; 2 Department of Psychiatry and Forensic Medicine, Universitat Autònoma de Barcelona (UAB), Barcelona, Spain; 3 Department of Psychiatry, Institut D’Investigació Biomèdica-Sant Pau (IIB-Sant Pau), Hospital de la Santa Creu i Sant Pau, Sant Antoni M. Claret, 167, 08025 Barcelona, Catalonia Spain; 4 Networking Research Center On Mental Health (CIBERSAM), Madrid, Spain; 5 Networking Research Center On Neurodegenerative Diseases (CIBERNED), Madrid, Spain

**Keywords:** Cognitive ageing, Neuroscience, Psychology, Risk factors, Signs and symptoms, Neurology, Dementia, Neurodegenerative diseases

## Abstract

Neuropsychiatric symptoms (NPS) have been recently addressed as risk factors of conversion to Alzheimer’s disease (AD) and other dementia types in patients diagnosed with Mild Cognitive Impairment (MCI). Our aim was to determine profiles based on the prominent NPS in MCI patients and to explore the predictive value of these profiles on conversion to specific types of dementia. A total of 2137 MCI patients monitored in a memory clinic were included in the study. Four NPS profiles emerged (classes), which were defined by preeminent symptoms: Irritability, Apathy, Anxiety/Depression and Asymptomatic. Irritability and Apathy were predictors of conversion to dementia (HR = 1.43 and 1.56, respectively). Anxiety/depression class showed no risk effect of conversion when compared to Asymptomatic class. Irritability class appeared as the most discriminant neuropsychiatric condition to identify non-AD converters (i.e., frontotemporal dementia, vascular dementia, Parkinson’s disease and dementia with Lewy Bodies). The findings revealed that consistent subgroups of MCI patients could be identified among comorbid basal NPS. The preeminent NPS showed to behave differentially on conversion to dementia, beyond AD. Therefore, NPS should be used as early diagnosis facilitators, and should also guide clinicians to detect patients with different illness trajectories in the progression of MCI.

## Introduction

Mild Cognitive Impairment (MCI) is a transitional stage between cognitively healthy aging and dementia, mainly Alzheimer’s Disease (AD)^1,2^. Since it is a heterogeneous nosological entity, several clinical subtypes of MCI have been described. According to cognitive performance, MCI can be classified into four groups: amnestic single (aMCI-sd) and multiple domains (aMCI-md), and non-amnestic single (naMCI-sd) and multiple domains (naMCI-md)^1^. The cognitive domains that may be affected include attention, memory, language, praxis, visuoperception, executive functions and visuospatial skills^3,4^.

Conversion rate to dementia for patients diagnosed with MCI is a controversial topic given that estimations of prevalence and incidence of dementia depend on multiple factors^5,6^. Among them, neuropsychiatric symptoms (NPS) have been postulated to be related to conversion. Indeed, NPS are highly prevalent in the majority of patients with dementia over the course of the disease^7^. In this context, some authors have pointed out NPS as being specific risk factors of conversion to dementia^8,9^. A recent update emphasized the importance of NPS as diagnostic and prognostic markers^10^. The relevance of such studies relies on the fact that NPS may be present even before the appearance of a significant cognitive decline or even before alterations of patients daily functioning^11^. Two studies have already analysed in different population settings (from volunteers to MCI patients) the differential conversion rates to dementia depending on the presence of NPS. The study by Leoutsakos and colleagues identified four groups based on NPS (1: irritable; 2: depressed; 3: complex; and 4: asymptomatic) finding that the complex group had the higher hazard ratio of conversion (3.20, 95% CI 2.24–4.58) in comparison with the asymptomatic group. The other study by Forrester and collaborators found three groups of patients classified according to NPS (1: severe cluster; 2: affective cluster; and 3: asymptomatic). In comparison to asymptomatic patients, individuals in the severe cluster showed more than twice the hazard of progression to dementia (2.69, CI 1.12–2.70), whereas the affective cluster had one and a half times the hazard of conversion (1.79, CI 1.12–2.70)^12,13^. Another recent study addressed the impact of NPS in patients diagnosed with MCI, and concluded that the coexistence of certain symptoms, i.e., hyperactivity, affect disturbances and psychosis, yielded conversion to dementia^14^. These findings point out the need to establish NPS profiles rather than exploring individual symptoms that may account for conversion outcomes.

However, the mere existence of NPS alone should not be considered the unique factor to determine the conversion from MCI to specific types of dementia. Age, gender or even, level of education may also account for the progression of MCI towards dementia^15,16^. In terms of neurobiological factors, apolipoprotein E epsilon4 (*APOE-Ɛ4*) has been found to be the main genetic risk factor for Alzheimer’s disease (AD), specifically with sporadic and late-onset forms^17–19^. Interestingly, a synergistic interaction between some NPS (depression or apathy) and *APOE-Ɛ4* has been found to increase the risk of dementia^20,21^. However, the possible influence of the *APOE-Ɛ4* on the relation between comorbid NPS and conversion to dementia in MCI patients has never been explored.

In light of the above arguments, it can be postulated that NPS may determine the progression from MCI to specific types of dementia. Therefore, the objectives of this study are (1) to explore consistent classes of NPS among patients with MCI using Latent Class Analysis (LCA); (2) to determine the effect of the resulting NPS classes on progression to dementia by means of a survival analysis; and (3) to investigate conversion to different types of dementia based on NPS classes accounting also for factors such as age, gender, level of education and/or *APOE-Ɛ4.*

## Results

Table 1 shows demographic and clinical characteristics of the final sample. The most prevalent NPS (measured with the NPI-Q) were depression (n = 1298, 60.7%) and anxiety (n = 1286, 60.2%), closely followed by apathy (n = 990, 46.3%), irritability (n = 832, 38.9%) and sleep disorders (n = 686, 32%). The least prevalent symptoms were appetite disorders (n = 188, 8.8%), disinhibition (n = 75, 3.5%) and agitation (n = 73, 3.4%).

**Table 1:** Demographic and clinical characteristics of the study participants (n = 2137).

	Mean (SD)
Age (yrs)	74.6 (8.2)
Gender (% of females)	58.5
Education (yrs)	7.3 (3.9)
MMSE (total score)	26.9 (1.7)
NPI-Q (mean of total symptoms)	4.19 (1.9)
Years of follow-up mean/median (range)	2.24/1.79 (.5–9.38)

*SD* standard deviation, *MMSE* mini-mental state examination, *NPI-Q* neuropsychiatric inventory questionnaire.

The final LCA solution was determined according to parameters included in Table 2: Bayesian Information Criteria (BIC), entropy and Vuong–Lo–Mendell–Rubin ratio test (LRT) non-adjusted and adjusted LRT. Based on these criteria, the 4-class model was considered to fit best. In detail, although BIC value of the 3-class model was the lowest, entropy value was higher in the 4-class model, starting to decrease for the 5-class model, this latter not being statistically significant in terms of the adjusted LRT. See Table 2 for all measures of tested models.

**Table 2:** Summarized model statistics for two- to five-class solutions of the latent class analysis.

	Number of latent classes			
	2	3	4	5
BIC	15,940.1	15,833.9	15,847.8	15,838.1
Entropy	.66	.7	.77	.67
Vuong–Lo–Mendell–Rubin likelihood ratio test (LRT) *p* value	< .001	< .001	< .001	.182
Lo–Mendell–Rubin adjusted LRT *p* value	< .001	< .001	< .001	.186

**Figure 1:**
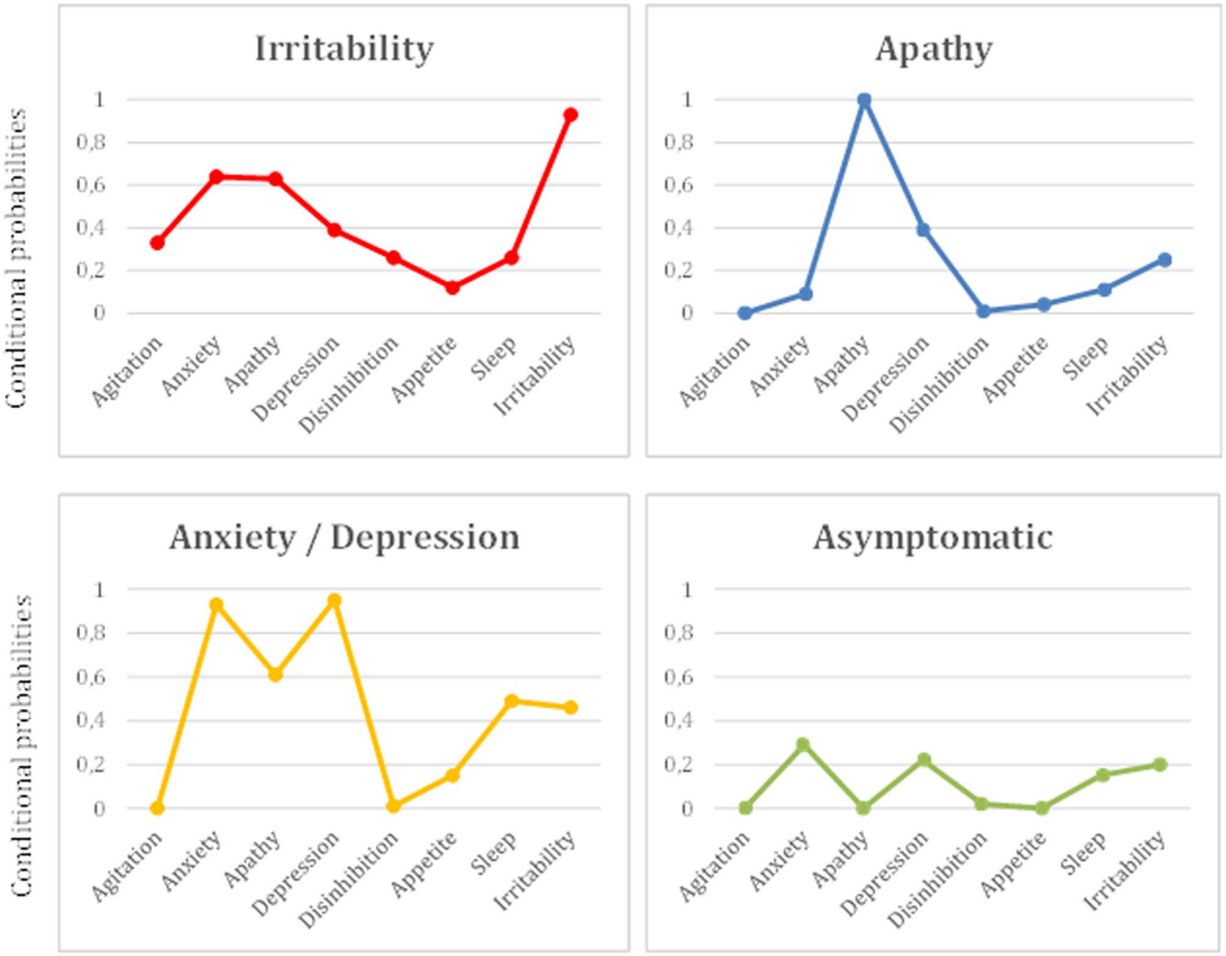
Profile plots represent estimated conditional probabilities (y-axis) observed in the latent class analysis (LCA) for the domains of the Neuropsychiatric Inventory-Questionnaire (NPI-Q; x-axis), displaying the 4-class solution: Irritability, Apathy, Anxiety/Depression and Asymptomatic.

The results revealed a structure in which each class was determined by specific symptomatology, and the most preeminent symptom was used to name every particular class (see Fig. 1). Class 1 (n = 134; 6.3%) was constituted by patients with high probability of irritability (.93), followed by far by anxiety (.64) and apathy (.63); Class 2 (n = 272; 12.7%) was strongly represented by apathy (1); Class 3 (n = 1056; 49.4%) showed high probability of depression (.95), anxiety (.93) and, by far, apathy (.61); and Class 4 included the rest of patients (n = 675; 31.6%) and was characterized by having low probabilities in all domains (< .3). Therefore, Class 1 was referred as ‘Irritability’, Class 2 as ‘Apathy’, Class 3 as ‘Anxiety/Depression’, and Class 4 as ‘Asymptomatic’.

Demographic and clinical variables stratified by clusters (4-class solution) are described in Table 3. There were significant differences among the four classes in most of the variables, with the exception of GDS. Regarding age, *Apathy* class was composed by the oldest patients, whereas *Anxiety/Depression* class was the youngest group. In relation to gender, *Irritability* class was predominantly composed by males, while patients classified in the *Anxiety/Depression* and *Asymptomatic* classes were mostly females. *Apathy* class showed the highest level of education, whereas the other 3 groups had similarly less years of education. In relation to general cognitive status, highest scores were found in the *Anxiety/Depression* class, followed by *Irritability* class. As for the NPS (presence/absence), taking into account the 12 domains present in the NPI-Q, *Anxiety*/*depression* class had the highest mean score in the number of symptoms suffered. The average length of follow-up was very similar among groups (over 2 years), with the exception of the *Apathy* class that showed the shortest length.

**Table 3:** Demographic characteristics of the study participants (n = 2137) stratified by the 4-class LCA model.

	Irritability class (n = 134)	Apathy class (n = 272)	Anxiety/depression class (n = 1056)	Asymptomatic class (n = 675)	F/*χ*^2^	*p*
Age (yrs)	75.17 (7.99)	76.20 (7.32)	73.82 (8.31)	75.23 (8.15)	8.30	**< .001**
Gender (% of females)	39 (29.1%)	112 (41.2%)	688 (65.2%)	411 (60.9%)	102.14	**< .001**
Education (yrs)	7.38 (3.80)	8.04 (4.23)	7.1 (3.79)	7.25 (4.08)	4.14	**.006**
MMSE	27 (1.72)	26.65 (1.74)	27.02 (1.69)	26.97 (1.76)	3.46	**.016**
GDS	3	3	3	3	.60	.600
NPI-Q (sum)	1.46 (1.35)	.58 (.79)	1.58 (1.27)	.35 (.63)	213.1	**< .001**
Years of follow-up mean/median (range)	2.31/1.79 (.52–8.62)	1.92/1.48 (.52–8.39)	2.27/1.78 (.50–9.38)	2.30/1.92 (.50–8.80)	4.02	**.007**
Conversion to dementia (%)	72 (53.7%)	149 (54.8%)	394 (37.3%)	264 (39.1%)	37.21	**< .001**
Time of conversion (median of years)*	2.69	2.09	3.30	3.55	21.01	**< .001**

Values represent mean (SD) or otherwise specified.

*MMSE* mini-mental state examination, *NPI-Q* neuropsychiatric inventory questionnaire.

*Kaplan Meier survival analysis.

Cox proportional hazard ratios were calculated to test three survival models of conversion to dementia. The first model only explored the effect of LCA on conversion; the second model was adjusted by age, Mini-Mental State Examination (MMSE), gender and years of education; and the third model included dichotomic *APOE-Ɛ4* status (0 = Non-carriers of 4 allele; 1 = Carriers of allele 4, either having one or two alleles) together with the factors of the second model. This latter model was executed in a subsample of patients, since not all patients had been genotyped for *APOE-Ɛ4* (n = 1106, 51.7% of the total sample). Probability of conversion to dementia in the subsample was 1.7 times more frequent between *APOE-Ɛ4* carriers than in non-carriers (χ^2^ = 15.4, *p* < .001), but the distribution of *APOE-Ɛ4* carriers was homogeneous between the 4 class groups (χ^2^ = .14, *p* = .99). Results from the three models are summarized in Table 4.

**Table 4:** Risk of conversion to dementia by 4 classes of preeminent neuropsychiatric symptoms applying three different models (Cox proportional hazards).

	Model 1 (n = 2137)	Model 2 (n = 2137)	Model 3 (n = 1106)
Irritability class	1.35 (1.04–1.75)*	1.43 (1.09–1.86)*	1.5 (1.07–2.08)*
Apathy class	1.70 (1.38–2.07)**	1.56 (1.28–1.92)**	1.43 (1.1–1.85)**
Anxiety/depression class	.96 (.82–1.12)	1.14 (.97–1.33)	1.08 (.89–1.31)
Asymptomatic class	Reference class	Reference class	Reference class

Values are hazard ratios (CI 95%).

Model 2 = Adjusted by class group, age, gender, MMSE score, and years of education. Model 3 = Adjusted by class group, age, gender, MMSE score, years of education and APOE-Ɛ4.

*p < .05; **p < .001; Model 1 = Adjusted by class group.

**Figure 2:**
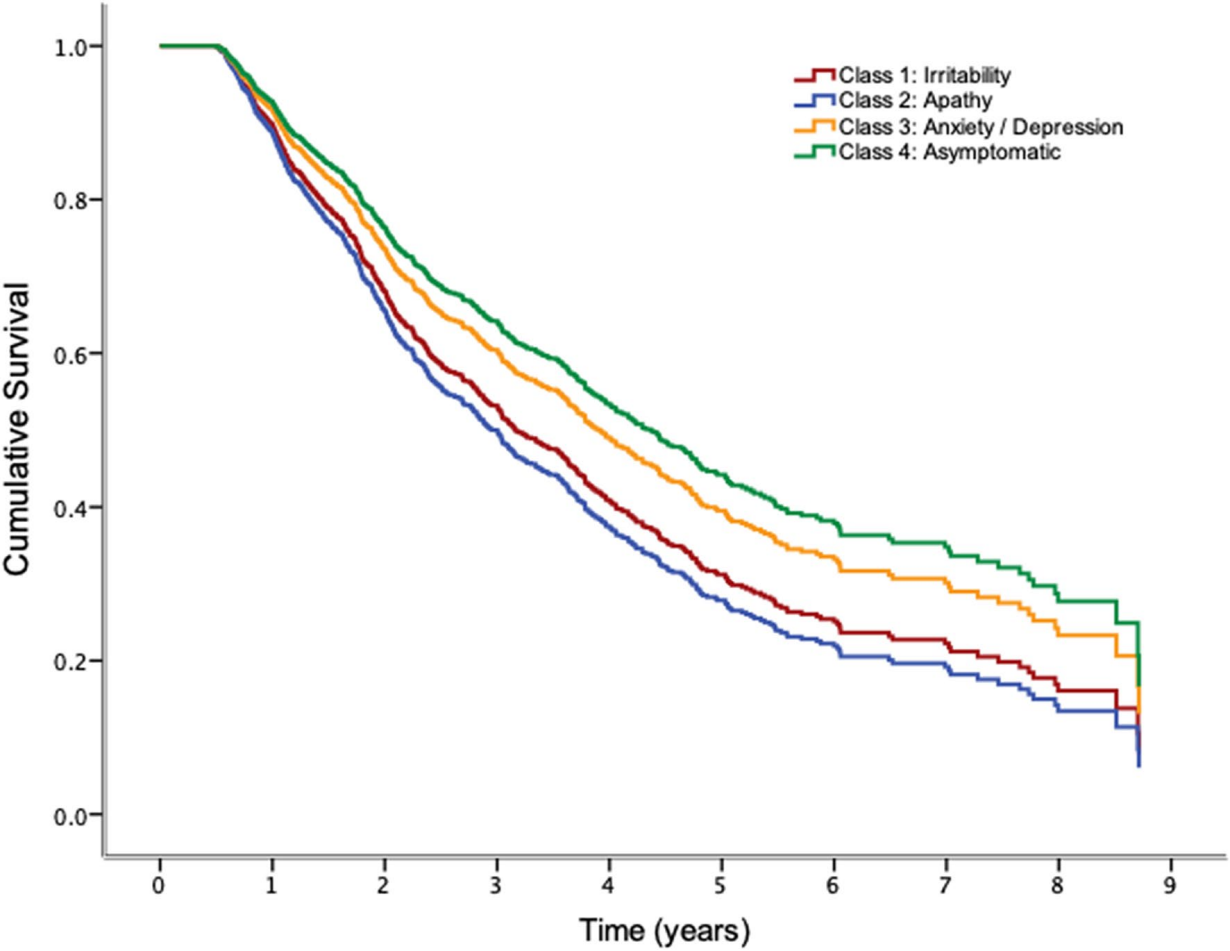
Survival curves of model 2 (adjusted by age, gender, mini-mental state examination and years of education) of the 4-class model obtained with LCA. Irritability and apathy classes showed significant increased hazard risks to convert to dementia compared to asymptomatic class.

*Irritability* and *Apathy* classes showed a higher risk of conversion when compared to the *Asymptomatic* class (reference class). Moreover, this effect appeared to be independent of adjustment variables (age, education, MMSE and *APOE-Ɛ4)*. By contrast, pertinence to the *Anxiety/Depression* class did not seem to add an extra risk of conversion compared to the *Asymptomatic* class. Figure 2 shows survival curves for the 4-class groups in the adjusted model.

The total percentage of converters was 45% (see Table 5 for specific class conversion to dementia). Percentages of conversion to dementia by class were 58.2% of patients in the *Irritability* class (n = 78); 58.8% in the *Apathy* class (n = 160); 41.2% in the *Depression/Anxiety* class (n = 435); and 42.7% in the *Asymptomatic* class (n = 288). Patients classified into the irritable group had lower percentages of conversion to AD and higher for BvFTD than the rest of classes. For the total sample of converters, grouping AD versus other dementias revealed a significant association with neuropsychiatric classes (χ^2^ = 47.4; *p* < .005). When taking the *Asymptomatic* class as the reference condition, the hazard ratio of conversion to non-AD dementia was 5.6 times higher (*p* < .005) in the *Irritability* class, while in the *Apathy* and *Anxiety/Depression* classes this hazard was 2.6 and 1.99, respectively (*p* < .005). The risk of conversion to non-AD dementias was similar between *Apathy* and *Anxiety/depression* classes (*p* = .173), but patients belonging to the *Irritability* class presented a higher probability of conversion to non-AD dementias compared to individuals in the *Apathy* class (OR = 2.16, *p* < .015).

**Table 5:** Specific conversion type of dementia for 4 classes of preeminent neuropsychiatric symptoms.

	AD	BvFTD	VD	PD	DLB	DPD	Non-degenerative	Others
Irritability class (n = 72)	23 (31.9%)	13 (18.1%)	21 (29.2%)	6 (8.3%)	0 NA	4 (5.6%)	5 (6.9%)	0 NA
Apathy class (n = 149)	75 (50.3%)	12 (8.1%)	41 (27.5%)	11 (7.4%)	4 (2.7%)	3 (2.0%)	3 (2.0%)	0 NA
Anxiety/depression class (n = 394)	224 (56.9%)	24 (6.1%)	91 (23.1%)	19 (4.8%)	6 (1.5%)	22 (5.6%)	7 (1.8%)	1 (.3%)
Asymptomatic class (n = 265)	192 (72.5%)	12 (4.5%)	40 (15.1%)	5 (1.9%)	6 (2.3%)	9 (3.4%)	1 (.4%)	0 NA
Total	514 (58.4%)	61 (6.9%)	193 (21.9%)	41 (4.7%)	16 (1.8%)	38 (4.3%)	16 (1.8%)	1 (.1%)

*AD* Alzheimer disease, *BvFTD* behavioral variant of fronto-temporal dementia, *VD* vascular dementia, *PD* Parkinson’s disease, *DLB* dementia with lewy bodies, *DPD* dementia by psychiatric disorder, *Non-degenerative* dementia by non-degenerative disorders, *other* dementia by others. Only converters are included here.

## Discussion

Our study investigated the impact of NPS in the conversion to different types of dementia in a large cohort of MCI patients from a Memory Unit. The results of the LCA gave rise to four well characterized groups of MCI patients based on their NPS, i.e., *Irritability*, *Apathy, Anxiety/Depression* and *Asymptomatic* classes, which yielded different risk rates of conversion to dementia.

Those patients with MCI classified as ‘irritable’ (Class 1) tended to convert mainly to AD, but also to other different types of dementia (BvFTD and VD in similar percentages). Particularly, when analysing conversion to non-AD dementia, the *Irritability* class showed higher risk of conversion than the rest of symptomatic classes. By contrast, MCI patients belonging to the other NPS classes (*Apathy, Anxiety/depression and asymptomatic*) converted mainly to AD and, to a lesser extent, to VD, being both the most frequent types of dementia observed in our sample.

The survival curves of conversion to dementia showed on one hand a similar pattern for *Anxiety/Depression* and *Asymptomatic* classes, and on the other hand, *Apathy* and *Irritability* classes posed a risk factor of conversion to dementia contrary to the accepted fact of anxious and depressive symptoms being classically described to be associated with dementia in the long term^8,22,23^. These findings may suggest that early detection and an adequate classification of NPS could lead to better the management of MCI progression. It is true that some studies have also related clinical features associated with AD to be present in adults with no diagnostic of dementia but depression^24^. In this regard, it has been postulated that successful treatment of this low-mood related symptoms could ameliorate cognitive impairment, thus increasing the probability of reversion from MCI to normal cognition. Going further, some researchers have proposed to investigate whether maintained antidepressant treatment could improve performance on neuropsychological testing, even though the causes of the instability that characterizes MCI are not well defined yet^25^.

The most frequent MCI trajectory was conversion to AD dementia, followed by VD, mirroring epidemiological studies of AD prevalence^4^. Interestingly, percentages of conversion to different types of dementia significantly varied across NPS-defined classes. In particular, less than a third of MCI patients classified as ‘irritable’ converted to AD, while those with no NPS (*Asymptomatic* class) showed up to 75% conversion to AD. This finding sheds light on the importance of exploring NPS in the very early stages of dementia as it reveals a differential impact on the prediction to specific types of dementia, at least at a group level.

*Irritability* and, to a lesser extent, *Apathy* classes appeared to be the determining factors in the conversion to dementia in our MCI sample, and this has scarcely been described in the literature. Previous works already found that irritability was a relevant behavioural disturbance^11^, as well as apathy and agitation, with high rates of prevalence among MCI patients^26^. The unadjusted model indicated that *Apathy* class was the best predictor, while once adjusted (including *APOE-Ɛ4* and the rest of variables), *Irritability* emerged as the most relevant neuropsychiatric condition when predicting conversion to dementia. Previous works reported other neuropsychiatic symptoms and were carried out in different sample of individuals (healthy volunteers) where MCI could be incident rather than prevalent^12,13^. In any case, our results suggest that the preeminence of irritability should be taken into consideration provided that it may confer differential susceptibility to quicker decline and conversion to a variety of dementia types^27^, and highlight that a good characterization of MCI individuals is required, given the heterogenic nature of this diagnostic entity.

Most of the studies addressing the presence of NPS in aging and dementia have been mainly focused on anxious and depressive symptoms. For instance, Tau Ming Liew and colleagues have recently published a community-based study where these two symptoms were evaluated in order to analyse whether concurrence of both, associated with cognitive deficits, improved the specificity to identify subjects at high-risk for neurocognitive disorders. Their findings showed that the subtype with the highest risk of conversion to neurocognitive disorders was the group with both, NPS (anxiety/depression) and cognitive deficits^28^. Our findings show that, although anxiety and depression have been the most widely explored NPS in relation to MCI and dementia, other NPS are present in both stages and their nature can determine the prognosis of MCI. A possible explanation is that anxious and depressive symptoms may be more reactive, temporary and linked to the self-awareness of being cognitively and/or functionally affected. However, in light of our findings this is merely speculative, and could also be explained by the characteristics of the setting of the present study. According to our results, Sugarman and colleagues reported more mood symptoms and hyperactivity (such as irritability, agitation, etc.) to be associated with progression to AD, whereas treating depression was related to a higher probability of cognition improvement^25^.

Indeed, our findings may not be fully generalized to the MCI population, as prevalence and incidence differences between community samples and clinical settings have been described^29,30^. However, the present results highlight the existence of NPS and their undoubtable impact on MCI trajectories at a group level; yet the effect of NPS in the daily clinical practice remains to be clarified. A recent review has also shown that NPS predicted conversion to dementia, in which NPI-Q scores were higher in converters^31^. This represents an opportunity to think about potential interventions for the early stages of the different forms of dementia.

Our study has limitations that need to be acknowledged. The main weakness is that despite the fairly large sample, patients were followed up only for 2.2 years on average, which may not be sufficient time to determine full conversion rates. Longer longitudinal designs would allow observing whether the impact of NPS on conversion profiles is stable or evolves along time. Patients were evaluated through the NPI-Q to determine NPS, which may not capture other psychopathological symptoms reported in previous studies. In any case, the NPI-Q is one of the most commonly used scales in neurology units. Also, pharmacological treatment was not well characterized which could also flaw our results.

## Conclusions

The main finding of the present study is that patients diagnosed with MCI can also display NPS and such symptoms may lead to different MCI trajectories of conversion to dementia. In particular, ‘irritable’ patients tended to convert to non-AD dementia, while ‘apathic’, ‘anxious/depressed’ and asymptomatic individuals converted mainly to AD, even though these results can not be generalized to each and every individual case, but it may provide valuable information to clinicians about the probability of conversion to specific types of dementia in order to be aware. These results open a new venue in which an accurate assessment of NPS at the time of MCI diagnosis is to be considered mandatory, as the presence or absence of such symptoms may define the long-term outcomes. Finally, assessment of NPS may provide an invaluable information to establish treatment strategies aiming at slowing down the progression to dementia or at least to improve the quality of life of MCI patients along illness trajectory in the context of a Memory Unit.

## Methods

### Participants

To carry out the present study, a sample of patients with a baseline diagnosis of MCI was selected from the pool of patients at the Memory Clinic of *Fundació* ACE, Barcelona, Spain (see Sample Selection section for details of selection)^32^. Data was collected from January 2006 to June 2017.

### Diagnosis and procedure

Participants were referred to the Memory Clinic by their General Health practitioner due to cognitive problems (or subjective complaints) or by their own decision of being evaluated in the Open House Initiative of *Fundació ACE*. After recruitment, neurologists, neuropsychologists and social workers assessed all participants. Diagnoses were made via consensus in a daily clinical committee by those professionals. At baseline, our sample had the following characteristics: a Clinical Dementia Rating Scale (CDR) of .5; and a Global Deterioration Scale (GDS) of 3 at maximum. The diagnosis of MCI was based on the modified Petersen’s criteria for aMCI and naMCI, MCI-sd and MCI-md, and Lopez’s citeria for possible or probable MCI due to AD^33^; whereas for dementia diagnoses depending on the aetiology were made as it follows: AD diagnosis was based on NIA-AA criteria, diagnoses of the behavioural variant of frontotemporal dementia (BvFTD) were made using consortium criteria, Vascular Dementia (VD) was diagnosed following the NINDS-AIREN report, for dementia due to Parkinson’s disease (PD) the last published criteria by the Movement Disorders Society was used, for dementia with Lewy bodies (DLB) the fourth report of the DLB Consortium was followed, and dementia caused by a psychiatric disorder was diagnosed when there was a previous psychiatric disorder diagnosed by a professional and when no criterion for a neurodegenerative disease was met.

Baseline assessment and subsequent follow-ups were conducted following the same procedure at each visit. All participants were evaluated at baseline as indicated above, and each subsequent follow-up was carried out by the same professionals, who evaluated each case individually using collected information about current state in order to validate the appropriate diagnosis or to explore if any changes occurred in relation to conversion to dementia. In the event of any doubt, the case was discussed in the daily clinical committee for the reassessment of the diagnosis. Follow-ups were approximately done annually.

### Ethical considerations

Informed written consent was obtained from all participants. The referral center ethics committee (Hospital Clínic i Provincial of Barcelona) approved the patient recruitment and collection protocols, which were in accordance with ethical standards of the World Medical Association and the Declaration of Helsinki—Ethical Principles for Medical Research Involving Human Subjects.

### Measures

The Neuropsychiatric Inventory-Questionnaire (NPI-Q) is a simplified clinical scale used to assess dementia-related behavioural disturbances in 12 domains (delusions, hallucinations, agitation/aggression, depression/dysphoria, anxiety, elation/euphoria, apathy/indifference, disinhibition, irritability/lability, aberrant motor behaviour, sleep and nighttime behaviours, and appetite and eating disorders)^34^. In our study, NPI-Q was administered by trained physicians and the information about the patient was provided by a reliable informant (familiar or close others). For each of the 12 domains, a change during the last month was measured as present or absent (dichotomous variable). Psychometric properties of the NPI-Q are satisfactory, being the tests-retest correlations between total symptom and distress scores .80 and .94 respectively; interscale correlation between the NPI total score for all domains and the NPI-Q severity total was .91^35^.

### Sample selection

The present study included patients with MCI (N = 7118) diagnosed at the Diagnostic Unit of *Fundació ACE* (ACE). All participants were assessed by a neurologist, a neuropsychologist and a social worker. Diagnoses and reassessments were made via consensus by all the different professionals in a clinical committee as explained above^36,37^. In order to test the hypothesis of the study, a selection of subjects was defined by the following criteria: (1) at least one follow-up visit (n = 4645); (2) older than 44 years old (n = 3417); (3) a MMSE total score higher than 23 (n = 2793)^38^; (4) more than 6 months of follow-up (n = 2470); and (5) administration of NPI-Q at their basal visit (n = 2137), which requires presence of an informant. The final sample used for the present study was therefore of 2137 patients diagnosed as MCI.

### Analytical approach

LCA provides a flexible analytical approach that allows researchers to study patterns of observations in data and to make inferences about unobserved sources of population heterogeneity^39^. The strategy becomes a person-centred analytic tool focused on similarities and differences among people instead of relations among variables^40^. The main target of this strategy is to assemble participants sharing similar characteristics (person-centred approach) into distinct profiles, based on their expressions on a number of variables that are intercorrelated^41^. LCA uses patterns of responses on dichotomous variables to estimate two different parameters, called latent class probabilities and conditional probabilities. Latent class probabilities become prevalence of each class and conditional probabilities are rates of each analysed variable given membership in each latent class. Thanks to these estimations it is possible to have an individual probability of affiliation in every latent class, according to their pattern of symptoms and their modal class membership.

Firstly, dichotomous ratings on each of the 12 NPI-Q domains were obtained (0 = 0; 1 > 1–3). Aimed not to introduce noise in the LCA data processing, only neuropsychiatric conditions observed at least in the 3% of participants were included. Thus, Agitation/aggression (agitation), depression/dysphoria (depression), anxiety (anxiety), apathy/indifference (apathy), disinhibition (disinhibition), irritability/lability (irritability), sleep and night-time behaviours (sleep), and appetite and eating disorders (appetite) were the final domains included in our analysis. The final LCA model was determined using a consensus of several fit criteria. Lowest value of BIC^42^, Entropy value (a number close to one suggests a clear classification)^43^, LRT and adjusted LRT were performed to estimate whether a model with k profiles fitted the data significantly better than a model with k − 1 profiles^44^. An optimal application of LCA needs the consideration that variables included in the analysis are independent between them after conditional class membership is created. This assumption was tested using standardized bivariate residuals^45^, contrasting the observed symptom patterns to respect those predicted by the model. Once LCA was performed and the most parsimonious number of classes was determined, each participant was assigned to the class according the highest membership probability. Subsequently, Cox proportional hazards models, using the resulting latent class solution as main predictor, were executed in order to determine their survival effect on conversion to dementia. Given that not all patients had been genotyped for *APOE-Ɛ4*, comparability of this subsample with the sample without this measure was measured by means of χ^2^ contrasting the distribution for all NPS classes. All neuropsychiatric domains were statistically comparable between these subsamples. Lastly, in order to explore the frequency distribution of our four main variables of study (NPS-classes) we obtained a contingency table to ascertain different frequencies within type of dementia for each class. LCA was run using MPlus v8.4 and Cox analysis with SPSS V26.

## Data Availability

Data used for this study are available from the corresponding author on reasonable request.
